# Identification of two novel mutations on CLCN7 gene in a patient with malignant ostopetrosis

**DOI:** 10.1186/s13052-014-0090-6

**Published:** 2014-11-20

**Authors:** Giuseppe Bonapace, Maria Teresa Moricca, Valentina Talarico, Francesca Graziano, Licia Pensabene, Roberto Miniero

**Affiliations:** Department of Pediatrics, Laboratory of Molecular Biology, University Magna Graecia of Catanzaro, Catanzaro, Italy; Department of Pediatrics, Laboratory of Biochemistry, University Magna Graecia of Catanzaro, Catanzaro, Italy; Department of Pediatrics, University Magna Graecia of Catanzaro, Catanzaro, Italy

**Keywords:** Osteopetrosis, Clinical inconsistency, ATPase, Vacuolar proton pump, Splicing mutations

## Abstract

**Background:**

Osteopetrosis is a rare genetic disorder characterized by increased bone density due to a defective osteoclast’s bone resorption. Three clinical forms can be identified based on severity, age of onset and inheritance: the dominant benign form (ADO), the intermediate form (IRO) and the recessive severe form (ARO). Several genes have been involved in the pathogenesis of these different types of osteopetrosis. Many experimental evidences point out on a specific role for CLCN7, the gene encoding the chloride channel protein subunit alfa and for TCIRG1, the gene encoding an osteoclast specific subunit of the vacuolar proton pump. Mutations in CLCN7 gene have been associated to the complete spectrum of osteopetrosis ranging from ARO to IRO and even to ADO type II. On the other hand, mutations in TCIRG1 gene account for more than 50% of cases of ARO. It is then evident that the malignant osteopetrosis is characterized by a great molecular and clinical heterogeneity often making the final diagnosis difficult to achieve.

**Methods:**

We performed a complete clinical, biochemical and molecular analysis by PCR and direct sequencing, of a novel case of osteopetrosis with inconsistent clinical phenotype.

**Results:**

The patient, who cannot be ascribed to any of the ADO, ARO or IRO groups, carried two novel mutations in compound heterozygosis in the CLCN7 gene. The first was the missense mutation c. 948C > T on exon 10 that produces an Arg to Cys change, while the second was the IVS11 + 5G > A splicing mutation that resides on the donor splice site of intron 11 and distrupts the canonical splice site.

**Conclusion:**

Our data

- Demonstrate that the unusual clinical presentation observed in our patient with a mild clinical onset evolving towards a more serious clinical picture, is associated to two novel mutations on CLCN7 gene.
- Support the already described clinical and molecular heterogeneity of the malignant osteopetrosis
- Suggest that, performing a molecular diagnosis of osteopetrosis with inconsistent clinical presentation these two novel mutations have to be first considered.

## Background

Osteopetrosis is described as a heterogeneous group of diseases [1] related to defective bone resorption [2,3]. In the maintenance of this function, the regulation of pH in specific intracellular and extracellular compartments of osteoclasts is critical [4]. Beside the proton production, mainly due to the carbonic anhydrase II activity (CAII), a pivotal role is played by the mobilization of other ionic species. In particular, extrusion of chloride anions is required to neutralize the electric current generated by the H^+^-ATPase responsible for the acidification of the resorption compartment [5]. In this context a specific role both for the CLCN7 and TCIRGI genes has recently been demonstrated [6]. CLCN7 (Gene Bank NG_007567) is a member of the mammalian CLC gene family. It is expressed in different cell type in the vesicles of the endocyte-lysosomal pathway. In the osteoclast, the encoded protein clc7 (UniProt P51798) is localized in the ruffled border and it is involved in the acidification process. In humans, mutations in the CLCN7 gene give rise to a complete spectrum of malignant osteopetrotic phenotypes [7]. The protein encoded by the TCIRG1 gene, Tcrg1, (Gene Bank NG_007898,Uni Prot Q13488) is a subunit of the vacuolar proton pump involved in the process of extracellular acidification. This protein was initially identified in mice as produced by an osteoclast-specific gene, whose targeted deletion causes osteoclast-rich osteopetrosis [8]. In humans TCIRG1 is the most common affected gene accounting for about 50% of the malignant osteopetrotic phenotypes.

Clinically osteopetrosis is classified in three different forms: malignant infantile form with poor prognosis and autosomal recessive inheritance (ARO), benign/adult osteopetrosis with autosomal dominant inheritance (ADO) and autosomal recessive intermediate form (IRO) with clinical manifestation similar to the malignant form but with a lower incidence. Despite of this classification a clear genotype-phenotype correlation is not evident in the patients so far described. We report on the clinical and molecular characterization of a novel case of malignant osteopetrosis that for presentation and progression is not classifiable in any of the above mentioned clinical groups and demonstrate that the inconsistent phenotype in our patient is related to the presence of two novel mutations in the CLCN7 gene.

## Case report

The patient was a 16 months girl, born from non consanguineous parents referred to our department for a suspect of osteopetrosis. At the admission, the radiological investigation showed a small left wrist fracture due to trivial (minor) trauma, a diffuse increase in the bone density with obliteration of the marrow cavity and evidence of a bone appearance in the small bones of the hands and feet. The past medical history of the patient revealed she was hospitalized in the first month of life for fall to thrive, hepatosplenomegaly with increased sereum levels of lactic deidrogenase (LDH), creatin phosphokinase (CPK) alkaline phosphatase (ALP) and aminotransferases.

The patient showed a weight of 8,6Kg (5^-10^ percentile), a height of 76, 5 cm (25^ percentile) and a head circumference of 48 cm (<90^ percentile). Moderate hepatosplenomegaly and delayed dental eruption were also observed. The fundoscopic examen was negative. All the laboratory findings were normal except for a marked increase in the serum level of LDH (1850 UI), CPK (727 UI) ALP and aminotransferase. The urinary pH was 5,5. The hemogram was normal. The early onset of the carbonic anhydrase II deficiency syndrome is sometimes characterized by spontaneous fractures without renal acidosis [9]. Therefore based on the clinical and radiological evidences we evaluated the CAII activity that was in the normal range (15 U/mgHb) allowing us to rule out such condition. A blood sample was collected to perform a molecular study of the main genes involved in the malignant osteopetrosis.

Eight months later the patient was hospitalized again because of the worsening of the clinical conditions. Specifically the patient had a severe growth retardation, macrocephaly, frontal bossing, faillure of dental eruption, visual impairment with nystagmus, hemiparesis of left inferior limb without other cranial nerve’ involvement. Hepatosplenomegaly was increased.

**Figure 1:**
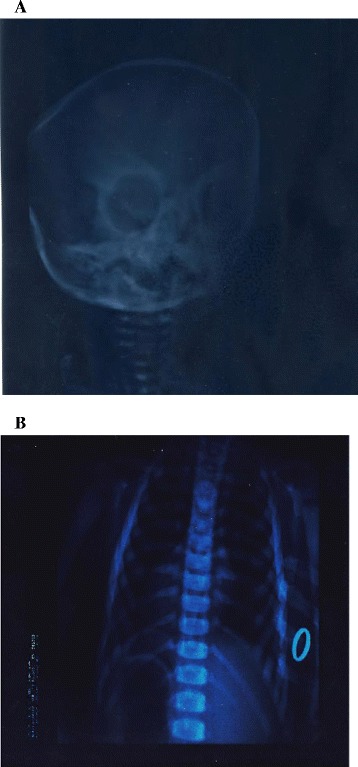
**Radiographic analysis of a case with CLCN7 dependent OP. A)** Skull view showing thickening of the inner and outer cortical tables. **B)** Chest showing generalised osteosclerosis in the thoracic cage.

The laboratory investigation revealed severe normocytic-normochromic anemia, trombocytopenia, persistent high levels of LDH, CK and serum acid phosphatase. Serum calcium was normal. The radiological skeletal survey confirmed the osteopetrotic picture observed before (Figure 1). The molecular and genetic evaluation demonstrated the involvement of CLCN7, one of the main gene responsible of malignant osteopetrosis. Based on all of the above described evidences a final diagnosis of malignant osteopetrosis with atypical onset was established. Because of the rapid clinical worsening, the patient underwent to a succesfull allogenic bone marrow transplantion (the oldest sister, resulting HLA full matching, was the donor. Specifically, visual impairment was completely rescued as well anemia, and trombocytopenia,. A normalization of LDH, CK and serum acid phosphatase levels was observed.

## Methods

### Sample blood collection

Informed consent was obtained from all subjects and/or their parents. All the procedures described above were performed for diagnostic purpose.

Blood specimens were collected by venipuncture in heparinized test tubes. After collection the specimens were diluited 1:100 with distilled water and then stored to -20°C until the hemoglobin and CAs were assayed.

### Hemoglobin assay

For the evaluation of Hb levels a spectrophotometric assay was used.

Briefly, 0.2 ml of 1:100 diluted blood were added to 0.8 ml of ammonium hydroxide solution 0.006 N. The optical density at 576 nm (OD 576) was read on a Beckmann DU 60 spectrophotometer using as blank a 0.2 ml of distilled water. The hemoglobin levels (mg/ml) were calculated by the formula: mg Hb/ml = OD576 × 500/0.912 [9].

### CAs activities

The CAI plus CAII (Ca_tot_) and CA II specific activities were evaluated according to the “Fish Tank Assay” method [9].

Briefly: for each patient two tubes with phenol red solution as indicator without or with 0.125 sodium iodide as inhibitor of CAI activity were prepared. The tubes were left on ice at 4°C for one hour. Carbonic anhidride (CO2) was bubbled in all tubes at 600 ml/min for 60 seconds, and then 100 μl of water for uncatalyzed reaction (Tuc) or a 1:100 diluition of sample blood for the catalyzed reaction (Tc), 2 ml of a barbital buffer (diethylbarbituric acid, ) ice cold pH 7.8 were added to start the reaction. The time required to reach the end point at pH 7.2 after the addition of barbital buffer, was measured using a standard phenol red solution pH 7.2 as indicator dye. The CA_tot_ (CAI plus CAII activities) and CAII specific activities were calculated by the expression: Tuc-Tc/Tc X diluition factor (where Tuc = Time of uncatalyzed reaction, Tc = Time of catalyzed reaction). Results for CA levels were expressed in units of CA per mg of Hb.

### Polymerase chain reaction

Blood samples for the molecular analysis, were drawn by venipuncture in EDTA pretreated tubes, After DNA extraction, all the exons and the boundary intronic regions were individually amplified by Polymerase Chain reaction (PCR) using nucleotide primer pairs designed on the basis of the published CLCN7 and TCIRG1 gene sequences. PCR amplification was performed using 100 ng of genomic DNA in a 30 μl volume with 250 μM final concentration of dNTPs, a 1.5 mM final concentration of MgCl2, 10 pM of of each primer and 1U Taq DNA polymerase. After heating 94°C for 3 min, 30 cycles were performed at 94°C for 1 min, 55°C for 1 min and 72°C for 1 min before a final step of 72°C for 10 min. Each PCR product (15 μl) was electrophoresed on 1.8% agarose and visualized using ethidium bromide staining.

### Direct sequencing

The PCR products were purified by PCR Purification Kit (Roche) and sequenced by dideossi chain terminator reaction using a Big-Dye terminator kit according to the manufacturer’s instructions (A.P.Biosystem) and run on 310 ABI Prism Analyzer The sequences were then analyzed using a FASTA and ASSEMBLING software (A.P. Biosystem). The point mutations identified were confirmed by sequencing the opposite strands and comparing with the relative wild type sequences published in gene data bank (CLCN7 Ref NG_007567; TCIRG1 Ref NG_007878. The putative structural consequences of missense mutations were predicted based on the sequences analysis and multiple sequence alignment information.

### Bioinformatic analysis

The effects of the putative novel splicing mutation (defined as never described on HGMD site www.hgmd.cf.ac.uk/ac/index.php or published in Pubmed) were studied in silico by using the ASSEDA software (http://splice.uwo.ca/cgi-bin/protected/display.cgi?forweb_sis).

## Results and discussion

### Carbonic anhydrase II assay

We ruled out a possible osteopetrosis due to a carbonic anhydrase II deficiency by performing the residual enzyme activity evaluation. According to the reference value for the human Carbonic anhydrase isoenzyme II activity already published, [9] we found in our patient a value of 15 U/mg of Hb in the normal range. This data allow us to exclude the Carbonic anhydrase II deficiency syndrome.

### Molecular analysis

**Figure 2:**
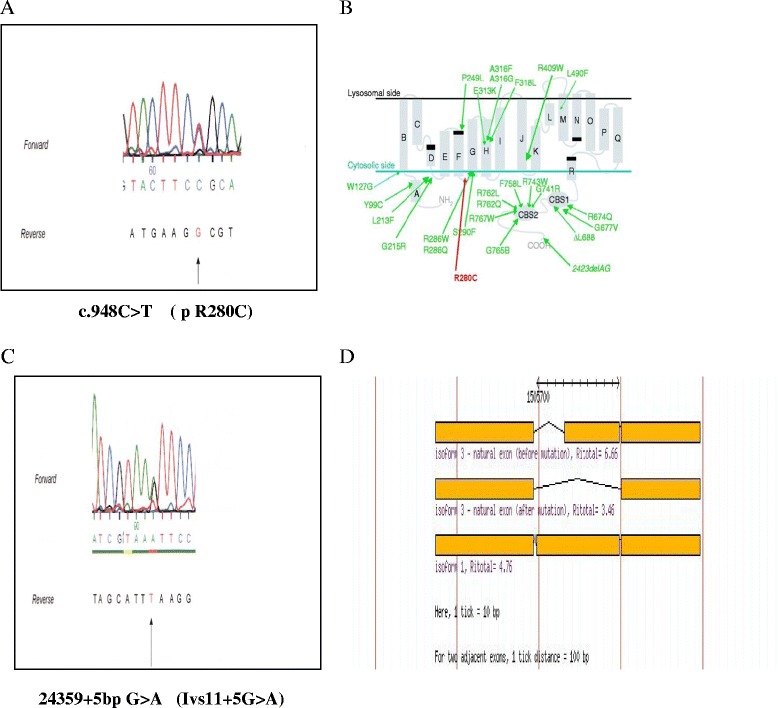
**Novel Mutations on CLCN7 gene in a case of OP with inconsistent phenotype. A)** Electrophoregram showing the c.948C > T transition on Exon 10 of CLCN7 gene. **B)** The c.948C > T produces a change of Arg 280 to Cys (R280C) on the cytosolic loop between the 5th and 6th alfa transmembrane idrofobic domains. **C)** Electrophoregram showing the 23459 + 5 bp G > A transition (Ivs11 + 5 bp G > A) located on the donor splice site of intron 11. **D)** Asseda bioinformatic analysis of the effect of the Ivs11 + 5 bp G > A transition: the Ri score, drops down from the highest values of 4.1 for wild type to 0.9 for the mutant with a predicted folding change of -9.1 According to this evaluation an abberrant splicing could take place leading to the skipping of the Exon 12 using an alternative donor splice site with a calculated Ri of 3.46.

Based on the clinical and biochemical findings we decided to perform a molecular analysis of the CLCN7 and TCIRG1 genes primarily responsible for the different forms of malignant osteopetrosis. Figure 2 shows the results of the CLCN7 gene sequencing. On the exon 10 of the CLCN7 gene a transition C/T at codon 948 (c948C > T) was found. A second transition G > A is evident 5 bp after the end of exon11 on the donor splice site of intron 11 (IVS11 + 5G > A ). The molecular analysis of the patient’s parents demonstrate that the c948C > T mutation was maternal while the IVS11 + 5G > A was inherited from the father (data not shown) The in silico study based on sequences analysis and multiple sequence alignment information (http://www.ebi.ac.uk/) clearly indicate that the c948C > T transition on Exon 10 of CLCN7 gene produces a change of Arg 280 to Cys on the cytosolic loop between the 5th and 6th alfa transmembrane hydrophobic domains. Based on the amminoacid changed (a polar positive Arg with a more reactive Cys) and on the involved domain, it is possible to hypothesize that this mutation can have some significant structural consequences. The ASSEDA software analysis performed on the second mutation on CLCN7 gene, clearly demonstrates that the IVS11 + 5G > A transition resides on the donor splice site of intron 11 gene and affects the donor splice site. In fact, the Ri score, defined as the probability of using the involved sequence, after a base change, as canonical donor splice site, calculated according to Roca et al [10] drops down from the highest values of 4.1 for wild type to 0.9 for the mutant with a predicted folding change of -9.1(data not sown) According to this evaluation a defective splicing could take place leading to the skipping of the Exon 12 using an alternative donor splice site with a calculated Ri of 3.46. Only the well known c166C > T (R56W) polymorphism was found on TCIRG1. None of the above described novel sequence variations was found in 100 healthy unrelated controls, ruling out the possibility of a population polymorphism. Taken together this data suggest that both the mutations on CLCN7 could have a significant functional impact on the resulting protein.

## Conclusion

Osteopetrosis is a rare genetic disorder characterized by increased bone density due to a defective osteoclast’s bone resorption. Three main forms can be identified based on severity, age of onset and inheritance: the dominant benign form (ADO) that is further divided in two subgroups ADO I and ADO II (termed also Albers-Schonberg disease), the intermediate form (IRO) and the recessive severe form (ARO). Mutation in several genes have been associated with the disease but a comprehensive classification based on the molecular findings is not still available and the relationship between genotype and phenotype remains elusive.

*ADO* is usually diagnosed in adolescence or in adulthood. Finding may include fracture due to minor trauma, osteomyelitis of the mandible or septic osteitis or osteoarthritis. Systemic symptoms are absent. Life expectancy is usually normal.

*IRO* form is characterized by intermediate severity between ADO and ARO. The onset is in the childhood and clinical findings may include spontaneous fracture for minor trauma. Mild anemia and moderate visual impairment secondary to optic nerve compression may be observed. Life expectancy is good.

Affected children with *ARO* usually present with severe disease within the first year of life. Most important symptoms include faillure to thrive, severe anemia or pancytopenia, recurrent infections, gross hepatosplenomegaly, abnormal craniofacial appearance, frontal bossing, compression of cranial nerves with blindness. Bone radiographs show sclerotic bone changes with a sandwich appearance of the vertebrae and “ bone-within bone” features, sclerosis of the skull base. The disease is fatal and only bone marrow transplantation may be effective.

Clinically our patient may not be classified neither as ARO, because the onset was delayed to the second year of life even though the severity of the disease was very suggestive, nor as IRO, because there was no match both for age of onset and severity. Moreover the ADO form was easily ruled out because the disease was not autosomal dominant with onset and symptoms not well matching with this classification. Based on the clinical, genetic and molecular findings our patient might represent a subtype of ARO form with late onset. We don’t know if the slow clinical progression we observed is related to the novel mutations found on CLCN7. Experiments are in progress to assess the functional effects of both mutations above described, with a main focus on the splicing defect. With this work we contribute to the molecular dissection of the CLCN7 deficient ARO and provide new insights into the molecular bases of the disease which can be exploited for the molecular diagnosis of malignant osteopetrosis with an inconsistent clinical history and a not clear phenotype.

## References

[1] Balemans W, Van Wesenbeeck L, Van Hul W (2005). A clinical and molecular overview of human osteopetroses. Calcif Tissue Int.

[2] Barvencik F, Kurth I, Koehne T, Sta’uber T, Zustin J, Tsiakas K, Ludwig CF, Beil T, Pesta J, Hahan M, Santer R, Supanchart C, Kornak U, Del Fattore A, Jentsk T, Teti A, Schultz A, Schinke T, Amling M: **CLCN7 and TCRG1 mutations differentially affect bone matrix mineralization in osteopterotic individuale.** *J of Bone Min Res* 2013, doi:10.1002/jbmr.2100. 10.1002/jbmr.2100 24108692

[3] Frattini A, Orchand PJ, Sobacchi C, Giliani, Abinum M, Mattsson JP, Feeling DJ, Anderson AK, Wallbrandt P, Zecca L, Notarangelo LD, Vezzoni P, Villa A (2000). Defects in TCRG1 subunit of the vacuolar proton pump are responsible for a subset of human autosomal recesseive osteopetrosis. Nat Genet.

[4] Superti Fuga A, Unger S, the Nosology Group of the International Skeletal Displasia Society (2007). Nosology and classification of genetic skeletal disorders: 2006 revision. Am J Med Gen.

[5] Kornak U, Schultz A, Friedrich W (2009). Mutations in the a3 subunit of the vacuolar H(+)-ATPase cause infantile malignant osteopetrosis. Hum Mol Genet.

[6] Kornak U, Kasper D, Bosi MR (2001). Loss of the CLC-7 chloride channel leads to osteopetrosis in mice and man. Cell.

[7] Campos-Xavier AB, Saraiva JM, Ribeiro LM, Munnich A, Cirmier-Daire V (2003). Chloride channel 7 (CLCN7) gene mutations in intermediate autosomal recessive osteopetrosis. Hum Genet.

[8] Susani L, Pancrazio A, Sobacchi C, Taranta A, Mortier G, Savarireyan R, Villa A, Orchard O, Vezzoni P, Albertini A, Frattini A, Pagani F (2004). TCRG1-dependent recesseive osyeopterosis: mutation analysis, functional identification of the splicing defects and in vitro rescue by U1 snRNA. Hum Mut.

[9] Sly WS, Hewett-Emmett D, Whte MP, Yu YS, Tashian RE (1983). Carbonic anhydrase II deficiency identified as the primary defect in the autosomal recesseive sindrome of osteopetrosis with renal tubular acidosis and cerebral calcification. Proc Natl Acas Sci USA.

[10] Yu T, Yu Y, Wang J, Yin L, Zhou Y, Ying D, Huang R, Chen H, Wu S, Shen Y, Fu Q, Chen E (2014). Identification of TCRG1 and CLCN7 gene mutations in a patient with autosomal receseive osteopetrosis. Mol Med Rep.

